# Supplementary material for: OpenMM 7: Rapid development of high performance algorithms for molecular dynamics
Source: PLoS Comput Biol. 2017 Jul 26;13(7):e1005659. doi: 10.1371/journal.pcbi.1005659 (PMC5549999; doi:10.1371/journal.pcbi.1005659)
Supplement: S1 Source Code — (ZIP) [file pcbi.1005659.s002.zip › OpenMM-7.0.1-Source/docs/api-python/generated/simtk.openmm.app.charmmpsffile.CharmmPsfFile.html]

CharmmPsfFile — OpenMM 7.0.1.dev-5e86c4f documentation


# CharmmPsfFile¶

*class* `simtk.openmm.app.charmmpsffile.``CharmmPsfFile`(*\*args*, *\*\*kwargs*)¶
:   A chemical structure instantiated from CHARMM files.

    This structure has numerous attributes that are lists of the elements of
    this structure, including atoms, bonds, torsions, etc. The attributes are

    > - residue\_list
    > - atom\_list
    > - bond\_list
    > - angle\_list
    > - dihedral\_list
    > - dihedral\_parameter\_list
    > - improper\_list
    > - cmap\_list
    > - donor\_list # hbonds donors?
    > - acceptor\_list # hbond acceptors?
    > - group\_list # list of nonbonded interaction groups

    Additional attribute is available if a CharmmParameterSet is loaded into
    this structure.

    > - urey\_bradley\_list

    The lengths of each of these lists gives the pointers (e.g., natom, nres,
    etc.)

    Examples

    ```
    >>> cs = CharmmPsfFile("testfiles/test.psf")
    >>> len(cs.atom_list)
    33
    >>> len(cs.bond_list)
    32
    ```

    `__init__`(*\*args*, *\*\*kwargs*)¶
    :   Opens and parses a PSF file, then instantiates a CharmmPsfFile
        instance from the data.

        |  |  |
        | --- | --- |
        | Parameters: | **psf\_name** (*str*) – Name of the PSF file (it must exist) |
        | Raises: | - `IOError` (*If file “psf\_name” does not exist*) - `CharmmPSFError` (*If any parsing errors are encountered*) – |

    Methods

    |  |  |
    | --- | --- |
    | `__init__`(\*args, \*\*kwargs) | Opens and parses a PSF file, then instantiates a CharmmPsfFile instance from the data. |
    | `createSystem`(params[, nonbondedMethod, ...]) | Construct an OpenMM System representing the topology described by the prmtop file. |
    | `deleteCmap`() | Deletes the CMAP terms from the CHARMM PSF |
    | `loadParameters`(parmset) | Loads parameters from a parameter set that was loaded via CHARMM RTF, PAR, and STR files. |
    | `setBox`(a, b, c[, alpha, unit, beta, unit, ...]) | Sets the periodic box boundary conditions. |

    Attributes

    |  |  |
    | --- | --- |
    | `ANGLE_FORCE_GROUP` |  |
    | `BOND_FORCE_GROUP` |  |
    | `CMAP_FORCE_GROUP` |  |
    | `DIHEDRAL_FORCE_GROUP` |  |
    | `GB_FORCE_GROUP` |  |
    | `IMPROPER_FORCE_GROUP` |  |
    | `NONBONDED_FORCE_GROUP` |  |
    | `UREY_BRADLEY_FORCE_GROUP` |  |
    | `boxLengths` | Return tuple of 3 units |
    | `boxVectors` | Return the box vectors |
    | `system` | Return the cached system class – it needs to be initialized via |
    | `topology` | Create an OpenMM Topology object from the stored bonded network |

    `__delattr__`¶
    :   x.\_\_delattr\_\_(‘name’) <==> del x.name

    `__format__`()¶
    :   default object formatter

    `__getattribute__`¶
    :   x.\_\_getattribute\_\_(‘name’) <==> x.name

    `__hash__`¶

    `__reduce__`()¶
    :   helper for pickle

    `__reduce_ex__`()¶
    :   helper for pickle

    `__repr__`¶

    `__setattr__`¶
    :   x.\_\_setattr\_\_(‘name’, value) <==> x.name = value

    `__sizeof__`() → int¶
    :   size of object in memory, in bytes

    `__str__`¶

    `loadParameters`(*parmset*)¶
    :   Loads parameters from a parameter set that was loaded via CHARMM RTF,
        PAR, and STR files.

        |  |  |
        | --- | --- |
        | Parameters: | **parmset** (*CharmmParameterSet*) – List of all parameters |

        Notes

        - If any parameters that are necessary cannot be found, a
          MissingParameter exception is raised.
        - If any dihedral or improper parameters cannot be found, I will try
          inserting wildcards (at either end for dihedrals and as the two
          central atoms in impropers) and see if that matches. Wild-cards
          will apply ONLY if specific parameters cannot be found.
        - This method will expand the dihedral\_parameter\_list attribute by
          adding a separate Dihedral object for each term for types that
          have a multi-term expansion

    `setBox`(*a*, *b*, *c*, *alpha=Quantity(value=90.0*, *unit=degree)*, *beta=Quantity(value=90.0*, *unit=degree)*, *gamma=Quantity(value=90.0*, *unit=degree)*)¶
    :   Sets the periodic box boundary conditions.

        |  |  |
        | --- | --- |
        | Parameters: | - **a** (*length*) – Lengths of the periodic cell - **b** (*length*) – Lengths of the periodic cell - **c** (*length*) – Lengths of the periodic cell - **alpha** (*floats, optional*) – Angles between the periodic cell vectors. - **beta** (*floats, optional*) – Angles between the periodic cell vectors. - **gamma** (*floats, optional*) – Angles between the periodic cell vectors. |

    `topology`¶
    :   Create an OpenMM Topology object from the stored bonded network

    `createSystem`(*params*, *nonbondedMethod=NoCutoff*, *nonbondedCutoff=Quantity(value=1.0*, *unit=nanometer)*, *switchDistance=Quantity(value=0.0*, *unit=nanometer)*, *constraints=None*, *rigidWater=True*, *implicitSolvent=None*, *implicitSolventKappa=None*, *implicitSolventSaltConc=Quantity(value=0.0*, *unit=mole/liter)*, *temperature=Quantity(value=298.15*, *unit=kelvin)*, *soluteDielectric=1.0*, *solventDielectric=78.5*, *removeCMMotion=True*, *hydrogenMass=None*, *ewaldErrorTolerance=0.0005*, *flexibleConstraints=True*, *verbose=False*)¶
    :   Construct an OpenMM System representing the topology described by the
        prmtop file. You MUST have loaded a parameter set into this PSF before
        calling createSystem. If not, AttributeError will be raised. ValueError
        is raised for illegal input.

        |  |  |
        | --- | --- |
        | Parameters: | - **params** (*CharmmParameterSet*) – The parameter set to use to parametrize this molecule - **nonbondedMethod** (*object=NoCutoff*) – The method to use for nonbonded interactions. Allowed values are   NoCutoff, CutoffNonPeriodic, CutoffPeriodic, Ewald, or PME. - **nonbondedCutoff** (*distance=1\*nanometer*) – The cutoff distance to use for nonbonded interactions. - **switchDistance** (*distance=0\*nanometer*) – The distance at which the switching function is active for nonbonded   interactions. If the switchDistance evaluates to boolean False (if   it is 0), no switching function will be used. Illegal values will   raise a ValueError - **constraints** (*object=None*) – Specifies which bonds or angles should be implemented with   constraints. Allowed values are None, HBonds, AllBonds, or HAngles. - **rigidWater** (*boolean=True*) – If true, water molecules will be fully rigid regardless of the value   passed for the constraints argument - **implicitSolvent** (*object=None*) – If not None, the implicit solvent model to use. Allowed values are   HCT, OBC1, OBC2, or GBn - **implicitSolventKappa** (*float=None*) – Debye screening parameter to model salt concentrations in GB   solvent. - **implicitSolventSaltConc** (*float=0.0\*u.moles/u.liter*) – Salt concentration for GB simulations. Converted to Debye length   `kappa’ - **temperature** (*float=298.15\*u.kelvin*) – Temperature used in the salt concentration-to-kappa conversion for   GB salt concentration term - **soluteDielectric** (*float=1.0*) – The solute dielectric constant to use in the implicit solvent model. - **solventDielectric** (*float=78.5*) – The solvent dielectric constant to use in the implicit solvent   model. - **removeCMMotion** (*boolean=True*) – If true, a CMMotionRemover will be added to the System. - **hydrogenMass** (*mass=None*) – The mass to use for hydrogen atoms bound to heavy atoms. Any mass   added to a hydrogen is subtracted from the heavy atom to keep their   total mass the same. - **ewaldErrorTolerance** (*float=0.0005*) – The error tolerance to use if the nonbonded method is Ewald or PME. - **flexibleConstraints** (*bool=True*) – Are our constraints flexible or not? - **verbose** (*bool=False*) – Optionally prints out a running progress report |

    `system`¶
    :   Return the cached system class – it needs to be initialized via
        “createSystem” first!

    `boxLengths`¶
    :   Return tuple of 3 units

    `boxVectors`¶
    :   Return the box vectors

    `deleteCmap`()¶
    :   Deletes the CMAP terms from the CHARMM PSF

High performance molecular simulation on GPUs


### Navigation

- Application Layer
  - Loaders and Setup
  - Representation and Manipulation
  - Simulation
  - Reporting Output
  - Extras
- Library Layer

©2015, Stanford University and the Authors.
|
Powered by Sphinx 1.3.1
& Alabaster 0.7.7
|
Page source
